# Ultrasound versus nerve stimulation-guided interadductor approach for obturator nerve block: evaluation of injectate spread into the obturator canal in a randomized controlled trial

**DOI:** 10.1186/s12871-026-04008-2

**Published:** 2026-06-12

**Authors:** Tetsuya Uchino, Masahiro Miura, Chihiro Shingu, Toshitaka Shin, Kenichiro Tomonari, Shigekiyo Matsumoto

**Affiliations:** 1 Department of Anesthesiology, Faculty of Medicine, Oita University, 1-1, Idaigaoka, Hazama-machi, Yufu, Oita, 879-5593 Japan; 2 Department of Human Anatomy, Faculty of Medicine, Oita University, Oita, Japan; 3 Department of Urology, Faculty of Medicine, Oita University, Oita, Japan; 4 Oita Diagnostic Imaging Center, Oita, Japan

**Keywords:** Obturator nerve block, Interadductor approach, Ultrasound guidance, Nerve stimulation, Obturator canal, TUR-BT

## Abstract

**Background:**

This study aimed to compare the percentage of local anesthetic spread into the obturator canal between the nerve stimulation-guided interadductor approach (NS-INTAD) and the ultrasound-guided interadductor approach (US-INTAD), and to evaluate anatomical factors influencing the effectiveness of the obturator nerve block (ONB).

**Methods:**

Eighty patients scheduled for transurethral resection of bladder tumor were randomized to receive either NS-INTAD (*n* = 40) or US-INTAD (*n* = 40). In the lithotomy position, a mixture of local anesthetic and contrast medium was injected via the interadductor approach from the medial thigh. Injectate spread into the obturator canal was assessed by fluoroscopy, and the success rate and injection volume were compared between groups.

**Results:**

Enhancement in the obturator canal was observed in 75% of patients in the NS-INTAD group and 90% in the US-INTAD group. Although the percentage was higher in the US-INTAD group, the difference was not statistically significant (*P* = 0.139). The volume of local anesthetic used did not differ significantly between groups (*P* = 0.07). In patients where the posterior branch of the obturator nerve coursed between obturator externus muscle fascicles, local anesthetic propagation into the obturator canal appeared limited.

**Conclusion:**

The percentage of injectate spread into the obturator canal tended to be higher with US-INTAD than with NS-INTAD. Our findings suggest that the lithotomy-position approach facilitates more cephalad local anesthetic spread than the supine-position approach. Furthermore, the development of the superior fascicle of the external obturator muscle appears to be an anatomical factor that inhibits injectate spread into the obturator canal.

**Trial registration:**

UMIN Clinical Trials Registry (UMIN-CTR) UMIN000027762; registered 15 June 2017.

## Background

The obturator nerve traverses the pelvis and enters the obturator canal, where it bifurcates into anterior and posterior branches. After emerging from the thigh, the nerve exhibits considerable anatomical variability in its branching patterns [1–3]. In addition, hip branches may arise at various levels within the obturator canal, originating either from the main trunk or from its branches [4]. Consequently, simultaneous blockade of all obturator nerve branches, including the hip branches, requires diffusion of local anesthetic into the obturator canal [5, 6].

Cadaver studies have demonstrated pigment diffusion into both branches of the obturator nerve and within the obturator canal using a proximal approach for ultrasound-guided injection of local anesthetic between the pectineus and external obturator muscles [7–9]. However, a randomized controlled clinical trial demonstrated that when ≤ 10 mL of a mixture of local anesthetic and contrast medium was injected, successful obturator canal enhancement was achieved in fewer than 50% of cases using nerve stimulation-guided techniques (pubic and inguinal approaches), compared with 84% using the ultrasound-guided proximal approach [10]. Although obturator canal enhancement was observed in some cases with an injectate volume of approximately 5 mL, it was not achieved in others despite an injection volume of 10 mL. These results suggest that further improvement in injectate spread into the obturator canal during obturator nerve block (ONB) remains possible while maintaining a low injectate volume (≤ 10 mL).

On this basis, we focused on the interadductor approach, in which the needle is advanced from the medial thigh toward the exit of the obturator canal with the patient in the lithotomy position [5, 11]. This approach aligns the direction of needle advancement with the obturator canal exit. Moreover, leg elevation in the lithotomy position influences gravity, muscle tone, and tissue configuration, potentially enhancing the cranial spread of local anesthetic. The addition of ultrasound guidance enables precise injection into the intermuscular septum through which the nerve courses and is expected to further increase the anesthetic spread into the obturator canal.

In this study, we compared the percentage of injectate spread into the obturator canal between the nerve stimulation-guided interadductor approach (NS-INTAD) and the ultrasound-guided interadductor approach (US-INTAD). We also assessed procedure-related complications, such as vessel puncture and neuropathy, and compared our findings with previously reported the success rate of obturator canal enhancement for three ONB techniques [10].

## Methods

### Ethics

This single-center, blinded, randomized controlled trial was conducted at Oita University Hospital in Japan from August 25, 2017, when the first patient was enrolled, until September 27, 2019. Eighty-three patients scheduled for transurethral resection of bladder tumor (TUR-BT) were included. This study was approved by the Institutional Review Board of the Faculty of Medicine, Oita University, and was registered in the University Hospital Medical Information Network Clinical Trials Registry (Trial No. UMIN000027762; http://www.umin.ac.jp). All participants provided written informed consent prior to enrollment.

### Patient population

We enrolled patients aged 20 years or older with American Society of Anesthesiologists (ASA) Physical Status class I–III who were scheduled for TUR-BT under spinal or general anesthesia at Oita University Hospital, Japan, and required preoperative ONB at the urologist’s discretion. Exclusion criteria were serious hepatic or renal dysfunction, significant thyroid disease, allergies to local anesthetics or contrast agents, and pregnancy.

### Randomization and blinding

Eighty patients were randomly assigned in a 1:1 ratio to receive ONB via either the US-INTAD or NS-INTAD approach. Patient registration and allocation were performed using a web-based service with automatic randomization (https://mujinwari.biz/) employing a computer-generated block randomization method (block size of 4).

The assigned group was confirmed on a PC by an experienced anesthesiologist and the assistant immediately before performing the nerve block.

Participants were blinded to group allocation by shielding their lower limbs from view with an opaque drape during the block procedure. The anesthesiologist performing the block could not be blinded because of the nature of the interventions. Fluoroscopic images were assessed by a radiologist who was not involved in the block procedure and was blinded to group assignment. Statistical analyses were performed by an anesthesiologist not involved in the interventions.

### Anesthesia

For TUR-BT, spinal anesthesia was generally preferred. A total of 12–15 mg of 0.5% bupivacaine was injected at the L3–L5 levels using a 25-gauge, 7-cm Quincke needle (Spinocan; B. Braun, Melsungen AG, Germany). General anesthesia was administered to patients with coagulation abnormalities, a history of spinal surgery, or those who requested it. Induction was achieved with propofol and remifentanil, and anesthesia was maintained via a laryngeal mask (Proseal; Senko Medical Instrument Mfg. Co., Ltd., Tokyo, Japan) using oxygen, air, and either sevoflurane or desflurane. Anesthetic management, including ONB induction, was performed by the author (T.U.). During the nerve block procedure, patients’ feet were shielded from view using an opaque cloth placed at chest level. Concomitant perioperative care was provided according to routine clinical practice and did not differ between groups, including the type of anesthesia (spinal or general anesthesia) used for TUR-BT.

### Obturator nerve block (interadductor approach)

The ONB procedure was performed in the lithotomy position after induction of spinal or general anesthesia. A 22-gauge, 8-cm Teflon-insulated needle (Stimuplex D Ultra; B. Braun) was used. Detection of the obturator nerve was facilitated using a nerve stimulator (Stimuplex HNS 12; B. Braun) and an ultrasonic device (S-Nerve^®^; FUJIFILM-SonoSite Inc., Tokyo, Japan) with a linear probe (HFL 50; FUJIFILM-SonoSite Inc.). The ONB solution (10 mL of 1.2% mepivacaine) was prepared by adding 2 mL of iohexol to 8 mL of 1.5% mepivacaine. The ONB was performed by the same anesthesiologist (T.U.), and the assigned technique (NS-INTAD or US-INTAD) was delivered as allocated in all analyzed participants.

#### Nerve stimulation–guided interadductor approach (NS-INTAD)

The interadductor approach described by Kakinohana et al. was used as a reference [11]. The insulated needle was inserted 1 cm posterior to the long adductor tendon and 2 cm lateral to the pubic arch, then advanced with a slight lateral and posterior tilt. Stimulation began at 2–3 mA for 0.1 ms, and the current was gradually reduced while observing the motor response. Motor responses were assessed on the basis of the predominant adductor muscle contraction and its intensity. A small, focal contraction predominantly involving the adductor longus was considered to be suggestive of anterior-branch stimulation and was not used as the target response. Instead, a stronger adductor contraction (predominantly involving the adductor magnus, and/or simultaneous contraction of multiple adductor muscles) was used as the target motor response, consistent with stimulation of the posterior branch or the main trunk of the obturator nerve.

The needle was adjusted until stimulation recurred, and this process was repeated at 0.5–1 mA until a visible or palpable muscle twitch was achieved.

#### Ultrasound-guided interadductor approach with nerve stimulation (US-INTAD)

**Fig. 1:**
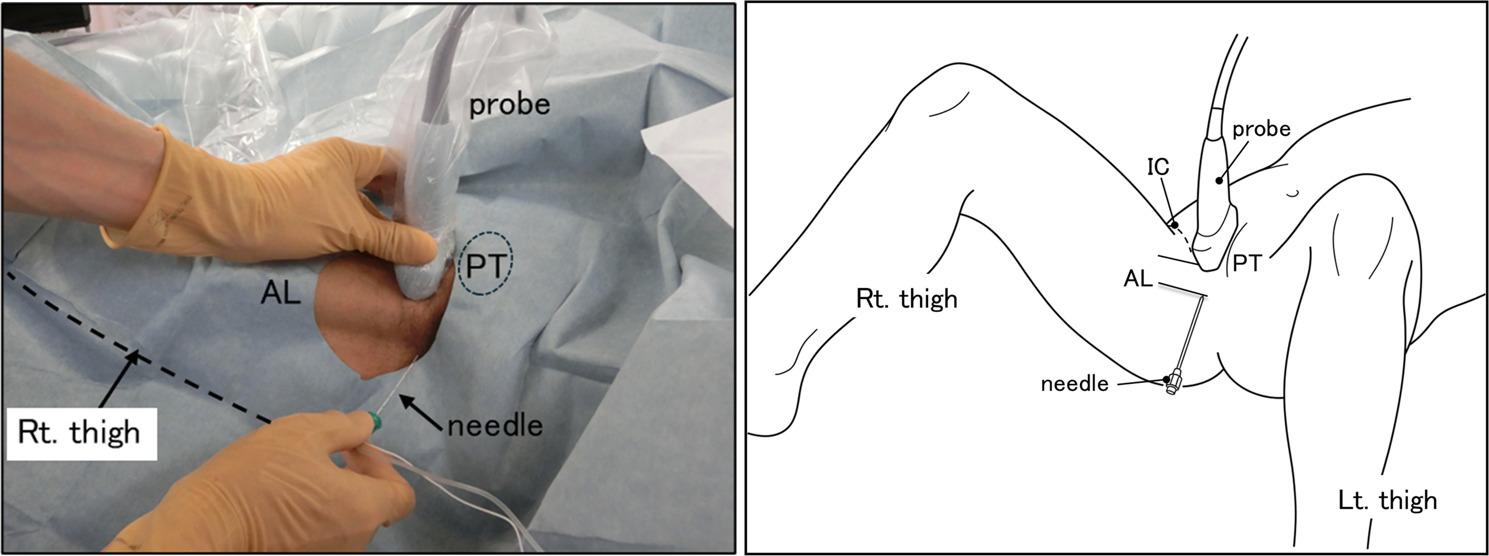
Patient and probe positioning during obturator nerve block using the ultrasound-guided interadductor approach. Left: Procedural photograph. Right: Schematic illustration of the same setup. The patient is placed in the lithotomy position with the legs abducted. A linear ultrasound probe is positioned over the right inguinal region adjacent to the pubic tubercle, with its long axis oriented nearly parallel to the long (cranio-caudal) axis of the trunk. The block needle is inserted in-plane along the long axis of the probe and is advanced from caudal to cranial. The skin entry site is posterior to the adductor longus tendon, and the needle passing deep to the adductor longus muscle. AL, adductor longus muscle; IG, inguinal crease; PT, pubic tubercle

**Fig. 2:**
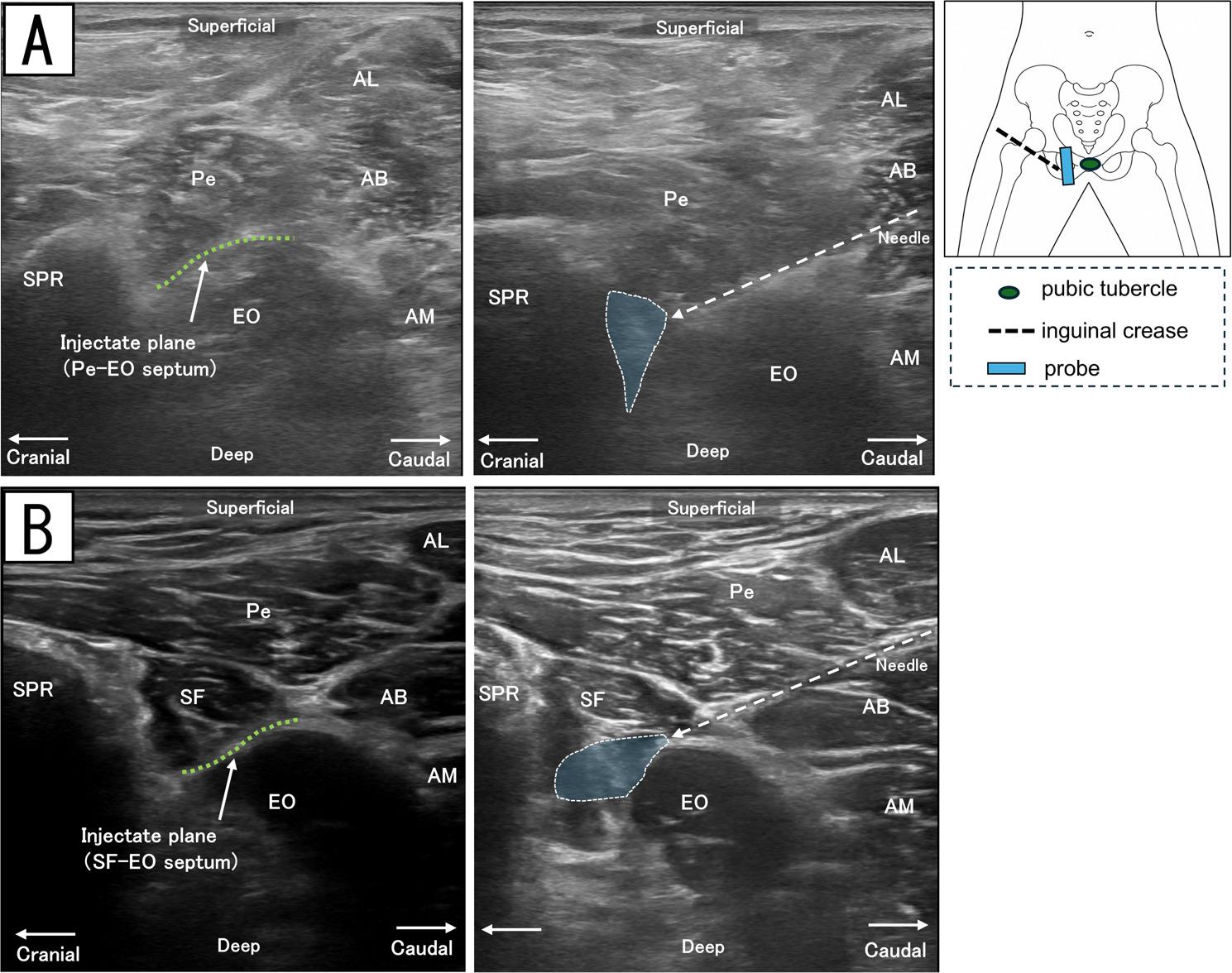
Ultrasound-guided interadductor approach for obturator nerve block. **A** No development of the superior fascicle of the external obturator muscle is observed. The needle is advanced in-plane toward the intermuscular septum between the pectineus muscle and the external obturator muscle. A mixture of local anesthetic and contrast medium is injected. AB, adductor brevis muscle; AL, adductor longus muscle; AM, adductor magnus muscle;Pe, pectineus muscle; EO, external obturator muscle; SPR, superior pubic ramus. **B** Development of the superior fascicle of the external obturator muscle is observed. The needle is advanced in-plane toward the intermuscular septum between the superior fascicle and the main body of the external obturator muscle. A mixture of local anesthetic and contrast medium is injected. AB, adductor brevis muscle; AL, adductor longus muscle; AM, adductor magnus muscle; Pe, pectineus muscle; SF, superior fasciculus of the external obturator muscle; EO, external obturator muscle; SPR, superior pubic ramus

The ultrasound probe was initially applied along the inguinal crease to visualize the thigh adductor muscle group and pectineus muscle. The probe was then rotated laterally toward the pubic bone by approximately 60–70° (clockwise on the right side and counterclockwise on the left side) to align the probe nearly parallel to the long (cranio-caudal) axis of the trunk (Fig. 1). The superior pubic ramus was first identified as a hyperechoic bony landmark, and the pectineus muscle was then recognized immediately superior to and adjacent to this landmark. The thigh adductor muscle group was subsequently visualized immediately caudal to the pectineus muscle. The external obturator muscle (EOM) was visualized deep to the pectineus muscle. The superior fascicle (SF) was defined as a distinct muscle bundle at the superior aspect of the EOM, separated from the main muscle belly by a linear hyperechoic band; when this bundle could not be clearly visualized, the corresponding side was classified as SF absent (Fig. 2).

When SF was not identified (Type A), the block needle was advanced to the intermuscular septum separating the pectineus muscle and the external obturator muscle (Fig. 2A). If SF was identified (Type B), the expected course of the posterior branch was inferred based on the intermuscular septum between the SF and the main body of the external obturator muscle, and the needle was directed toward this septum (Fig. 2B). Doppler imaging was then used to identify vessels around the target site. Neurostimulation was initiated at 2–3 mA with a 0.1 ms pulse width and was gradually decreased while monitoring for adductor magnus muscle contraction. Once the needle tip was confirmed by ultrasound to be within the intended intermuscular septum and adductor magnus contraction was elicited at 0.5–1.0 mA, the operator manually stabilized the needle to prevent migration during injection. The anterior branch was not separately identified or targeted. This strategy was based on prior evidence that the injectate deposited at the proximal/posterior-branch target plane can spread retrogradely into the obturator canal and surround both branches in a substantial proportion of cases [10]. The entire local anesthetic–contrast mixture was injected at a single target site without splitting the injection between anterior and posterior branches.

### Injection and fluoroscopy

**Fig. 3:**
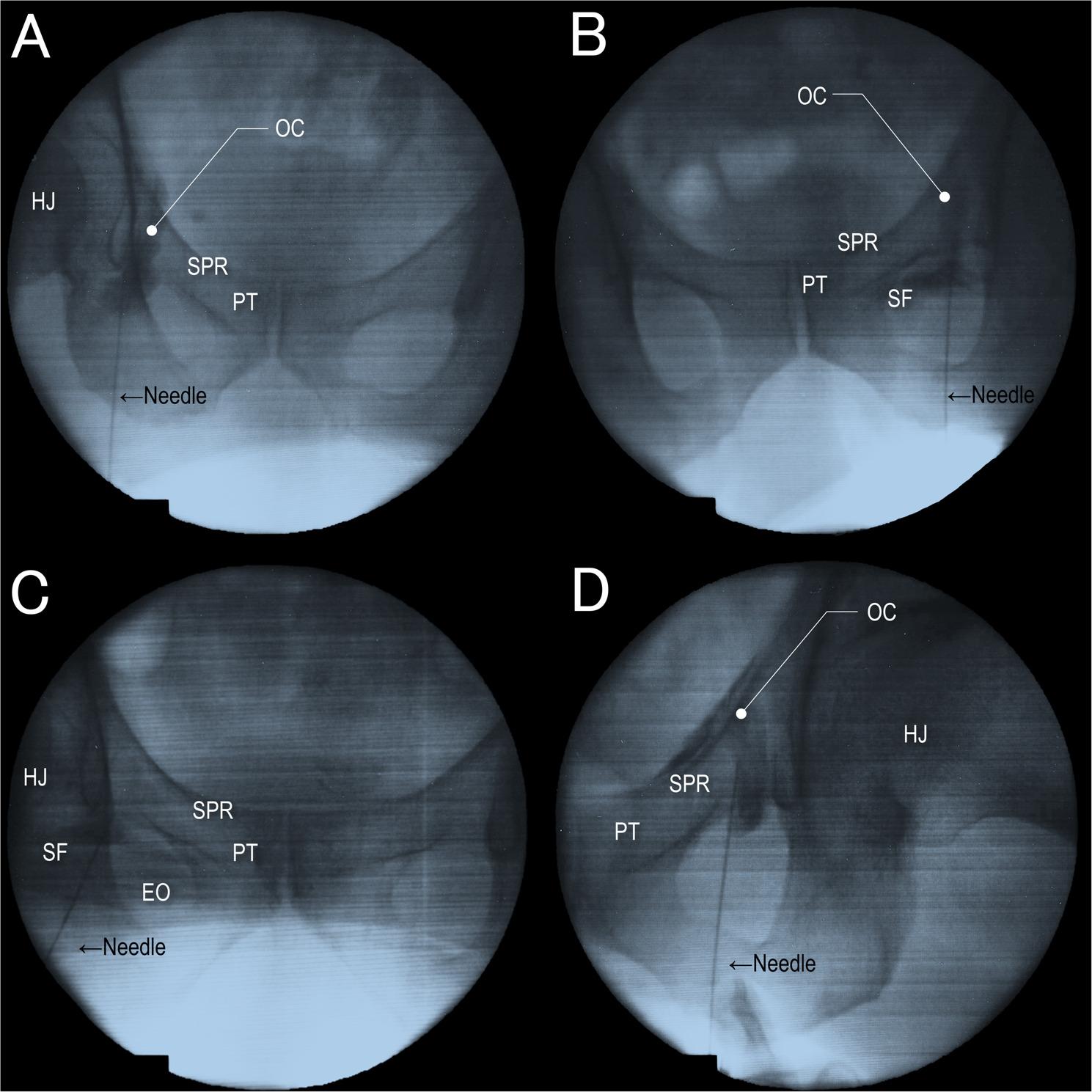
Contrast-enhanced images after obturator nerve block. Local anesthetic mixed with contrast medium is injected during the obturator nerve block, and the diffusion of the injectate is assessed under fluoroscopy. **A** The superior fascicle of the external obturator muscle is developed, and contrast medium is observed within the obturator canal. **B** The developed superior fascicle is enhanced, and contrast medium is observed within the obturator canal. **C** The developed superior fascicle and the main body of the external obturator muscle are enhanced; diffusion of contrast medium into the obturator canal is not confirmed. **D** A local anesthetic mixed with contrast medium was injected under fluoroscopy. The block needle was advanced deeply into the obturator canal using a nerve stimulation–guided interadductor approach. EO, external obturator muscle; HJ, hip joint; OC, obturator canal; PT, pubic tubercle; SF, superior fasciculus of the external obturator muscle; SPR, superior pubic ramus

After the block needle was fixed, 3 mL of the mixed solution was injected following aspiration to exclude intravascular placement. A radiologist (K.T.) evaluated all imaging findings. Initially, the diffusion of the contrast medium in the obturator canal was assessed by fluoroscopy after the 3 mL injection (Fig. 3). If 3 mL was insufficient to visualize the obturator canal, additional 1-mL increments were injected until the canal was visualized. Once visualization was achieved, injection was stopped, and the total injectate volume was recorded. If the obturator canal remained unvisualized after 10 mL, further injection was discontinued. Successful obturator canal enhancement was defined as visualization of the canal with ≤ 10 mL of solution.

Per Institutional Review Board requirements, the adductor reflex of the thigh was not to occur during surgery. Therefore, for patients in whom the obturator canal could not be confirmed by imaging, the needle tip was adjusted under fluoroscopic guidance, and additional solution was injected until adequate enhancement of the canal was achieved to prevent adductor reflex during TUR-BT.

Procedure-related complications were assessed during ONB, including intravascular injection or vascular puncture (based on aspiration and fluoroscopic contrast disappearance). Postoperative neuropathy was assessed clinically before discharge.

### Surgery

After fluoroscopic confirmation of the ONB, the urologist entered the operating room. During the procedure, electrical stimulation was applied to the bladder wall near the ureteral orifice.

### Sample size considerations

In a previous clinical study, the percentage of injectate spread into the obturator canal was 48% to 84% across three block techniques. Based on this, the success rates of both the nerve stimulation-guided technique and the new ultrasound-guided technique were assumed to be 60%, representing the null hypothesis. The alternative hypothesis assumed the nerve stimulation-guided method would remain at 60%, while the new ultrasound-guided technique would exceed the conventional method, reaching 90%. With a two-tailed significance level of 5% and a power of 80%, the required sample size was calculated as 36 participants per group. Therefore, the target sample size was set at 40 participants per group, for a total of 80 participants. No interim analyses were planned or performed, and there were no prespecified stopping guidelines.

### Statistical analysis

Statistical analyses were performed using SPSS version 29.0.2.0 (IBM Japan Ltd., Tokyo, Japan). The Shapiro–Wilk test was used to assess normality in the preliminary analysis. For comparisons among multiple independent groups, one-way ANOVA was used for height, while the non-parametric Kruskal–Wallis test was applied for age, body weight, and body mass index. For comparisons between the two groups, the Mann–Whitney U test was used for ASA physical status, and Fisher’s exact test was applied for types of anesthesia.

Obturator canal enhancement success rates among the two ONB techniques were compared using Fisher’s exact test. Comparisons of the number of failed attempts between the two groups were also analyzed using Fisher’s exact test. All tests were two-tailed, and *P* < 0.05 was considered statistically significant. No prespecified subgroup or sensitivity analyses were planned. A post-hoc exploratory analysis was performed to summarize the primary outcome according to ultrasound-defined anatomical types (Type A vs. Type B). Analyses were performed on a per-protocol basis, including only participants who received the allocated intervention and had evaluable primary outcome data. This study was reported in accordance with the CONSORT guidelines (see Additional file 1 for the completed CONSORT checklist).

## Results

### Study population and baseline characteristics

**Fig. 4:**
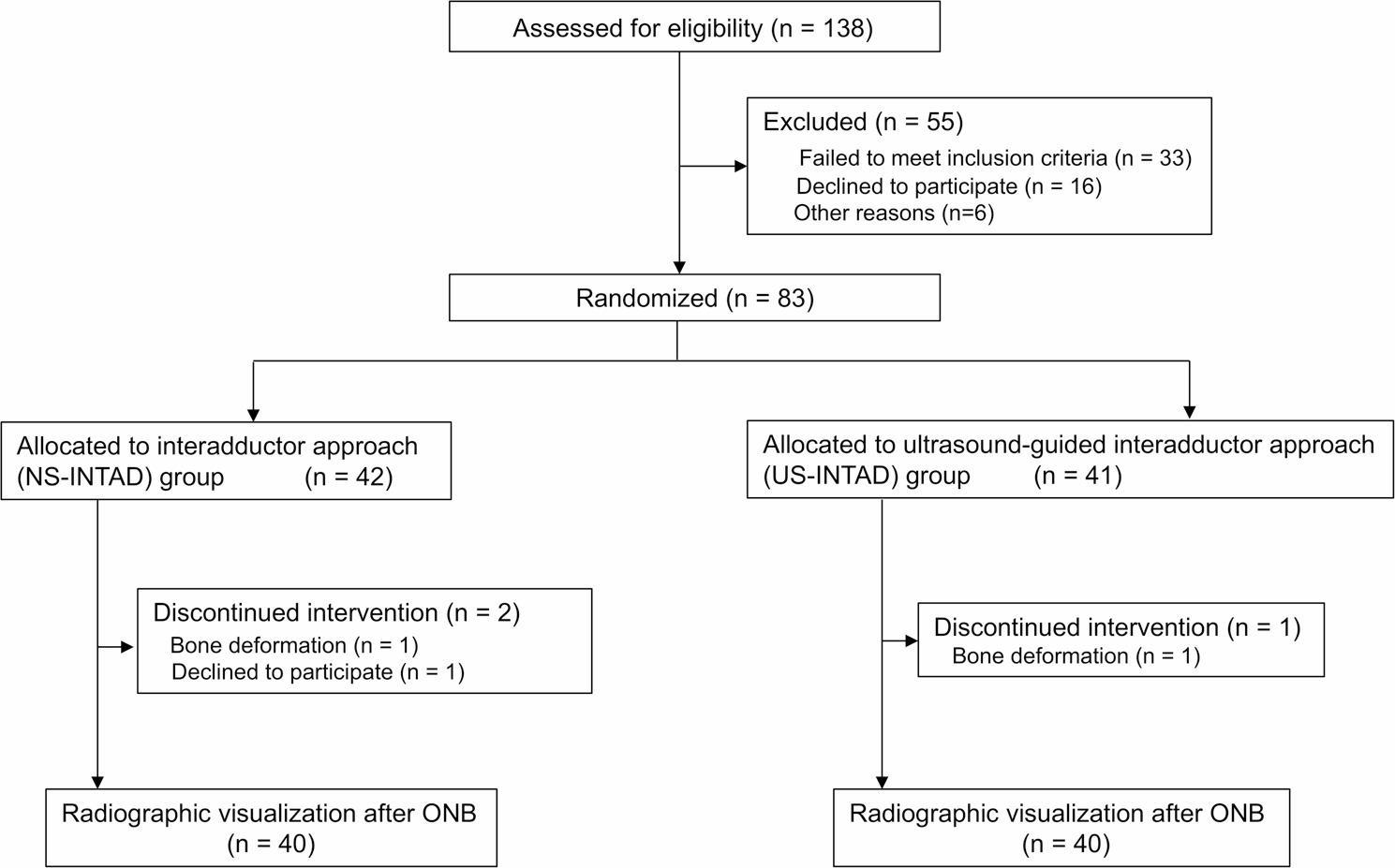
Study flow diagram

The study flow is summarized in Fig. 4, and baseline patient characteristics were comparable between groups (Table 1).

**Table 1:** Patient characteristics

		NS-INTAD	US-INTAD	*P*-value
Number of patients		40	40	-
Age [ yr ]		71.3 ± 9.1	68.2 ± 12.2	0.201
Height [ cm ]		164.6 ± 8.6	162.3 ± 7.6	0.225
Weight [ kg ]		66.8 ± 12.6	64.5 ± 12.2	0.403
BMI [kg/m^2^]		24.5 ± 3.5	24.4 ± 3.9	0.879
Anesthesia	Spinal	31(77.5%)	33(82.5%)	0.781
	General	9(22.5%)	7(17.5%)	
Gender	Male	38(95%)	34(85%)	0.263
	Female	2(5%)	6(15%)	
ASA physical status	ASA1	0	2(5%)	0.023
	ASA2	21(52.5%)	28(70%)	
	ASA3	19(47.5%)	10(25%)	

Data are presented as mean ± SD, median (range), or number (%)

*Abbreviations*: *ASA* American Society of Anesthesiologists, *BMI* body mass index, *NS-INTAD* nerve stimulation–guided interadductor approach, *US-INTAD* ultrasound-guided interadductor approach

### Primary outcome: obturator canal enhancement

Obturator canal enhancement was achieved in 30 of 40 patients (75%) in the NS-INTAD group and 36 of 40 patients (90%) in the US-INTAD group (risk difference, 15% points; 95% CI, − 8.9 to 36.2; *P* = 0.139) (Table 2).

**Table 2:** Comparison of the rates of successful obturator canal enhancement between the two groups

Group	Success	Failure	Success rate	*P*-value
NS-INTAD	30	10	75%	0.139
US-INTAD	36	4	90%	

*Abbreviations*: *NS-INTAD* nerve stimulation-guided interadductor approach, *US-INTAD* ultrasound-guided interadductor approach, *OC* obturator canal

As a post-hoc exploratory analysis, the primary outcome was summarized according to ultrasound-defined anatomical types (Type A vs. Type B) in the US-INTAD group. In the NS-INTAD group, the enhancement of the SF of the external obturator muscle was observed in 10 cases, whereas enhancement in the obturator canal was not achieved in any of these cases. In the US-INTAD group, 15 patients were classified as Type A and 25 as Type B. All 15 Type A cases exhibited enhancement in the obturator canal (Fig. 3A). Among the 25 Type B cases, 21 demonstrated obturator canal enhancement (Fig. 3B), whereas in the remaining 4 cases, contrast enhancement occurred in the SF of the external obturator muscle without reaching the obturator canal. (Fig. 3C)

### Secondary outcomes: injectate volume and complications

Among patients with successful obturator canal contrast enhancement, the mean injectate volume was 3.1 ± 0.5 mL in the NS-INSTAD group and 3.4 ± 0.6 mL in the US-INSTAD group, with no significant difference between the groups (*P* = 0.07). In the NS-INSTAD group, the block needle was advanced deeply into the obturator canal in eight patients; however, no intravascular injection was observed (Fig. 3D). In two of these patients in the US-INTAD group, although the aspiration test revealed no blood backflow, the immediate disappearance of the contrast after injection revealed intravascular migration. Therefore, the injection was halted, and needle reposition was repeated. No local anesthetic intoxication associated with intravascular injection of local anesthetic was observed. No postoperative neuropathy occurred in either group.

## Discussion

This study compared the percentage of local anesthetic spread into the obturator canal between the NS-INTAD and the US-INTAD. Obturator canal enhancement was achieved in 75% of patients in the NS-INTAD group and 90% in the US-INTAD group, indicating a higher success rate with US-INTAD; however, the difference was not statistically significant. The volume of local anesthetic and the incidence of complications were comparable between groups, and no safety concerns were identified.

With conventional nerve stimulation techniques, less than 50% of the injectate reaches the obturator canal [10], and this limited diffusion has been recognized as a clinical limitation. In contrast, the NS-INTAD group in this study achieved a 75% obturator canal spread, exceeding rates reported in previous studies. This improvement may be attributable to the interadductor approach, in which the needle trajectory is anatomically aligned toward the exit of the obturator canal, thereby facilitating injectate diffusion compared with the classical approach. However, in the NS-INTAD group, the block needle advanced deeply into the obturator canal in eight patients (Fig. 3D). Accordingly, when performing NS-INTAD, clinicians should remain mindful of the potential risk of vascular or adjacent organ injury associated with deep needle advancement.

For the ultrasound-guided proximal approach, local anesthetic injection between the pectineus muscle and the external obturator muscle was first described and confirmed in cadaver studies to stain both the anterior and posterior branches of the obturator nerve as well as the obturator canal [7–9, 12]. In the presented study, we hypothesized that using a robust obturator nerve motor response (predominantly involving adductor magnus muscle) as a procedural landmark in both groups would facilitate the spread of the injectate into the obturator canal. Consistent with this hypothesis, in all Type A cases, in which the posterior branch courses between the pectineus and external obturator muscles, a small volume of the mixed solution deposited in this plane spread into the obturator canal (Fig. 3A). In Type B cases, in which the posterior branch courses between SF and the external obturator muscle, the mixed solution was injected into the inter-fascicular septum traversed by the posterior branch. Notably, the injectate spread into the obturator canal in 90% of cases. In both groups, when contrast enhancement in the obturator canal was not achieved, the superior fascicle overlying the superior aspect of the obturator canal outlet was opacified. This suggests that the development of this fascicle may substantially impede injectate spread into the obturator canal. In this study, US-INTAD achieved obturator canal enhancement in 90% of patients with a small volume of injectate, exceeding the success rate reported for the ultrasound-guided methodological approach at 84% [10]. One contributing factor may be knee flexion during the block. Buyukfirat et al. reported that, in ultrasound-guided ONB, knee flexion improved nerve visibility and facilitated access because landmarks near the obturator canal were closest to the skin surface [13]. Similarly, in US-INTAD, knee flexion allowed an easier approach to the target intermuscular septum, likely enhancing injectate spread into the obturator canal. Another factor is leg elevation. Yoshida et al. demonstrated, in a cadaver study using an ultrasound-guided proximal approach in the lithotomy position, that the injected dye passed retrogradely through the obturator canal and stained both anterior and posterior branches of the nerve [7]. Gravity and altered muscle alignment from leg elevation may have further promoted cranial diffusion of the injectate.

Recently, new approaches directly targeting the obturator canal exit have been reported. Yoshida et al. demonstrated that ultrasound-guided ONB, which punctures the external opening of the obturator canal, allows local anesthetics and dyes to diffuse retrogradely into the pelvis and stain the entire obturator nerve [14]. Similarly, Labandeyra et al. observed a high rate of staining of the anterior branch and trunk of the obturator nerve using the obturator canal block [15]. Combined with the findings of this study, these results suggest that directly targeting the external opening of the obturator canal may be a promising strategy to ensure effective anesthetic spread, warranting further investigation. Moreover, in two patients in the US- INTAD group, intravascular uptake was detected. Although the aspiration test revealed no backflow of blood, intravascular placement was identified only by the rapid fluoroscopic disappearance of contrast, indicating that Doppler examination and the aspiration test may be insufficient to detect vascular puncture during US-INTAD.

This study has several limitations. First, as a single-center study with a limited sample size, the generalizability of the results is restricted. Second, potential bias cannot be excluded because the anesthesiologist performing the nerve block could not be fully blinded due to procedural constraints. Third, in patients with inadequate obturator canal enhancement after ONB, additional injections were administered under fluoroscopic guidance to prevent adductor reflex during surgery, making it impossible to accurately assess reflex development in these cases. Finally, individual anatomical variations and the development of the superior fascicle may have influenced drug diffusion, but the study design did not allow for a comprehensive analysis of these morphological differences.

## Conclusion

The ultrasound-guided interadductor approach achieved a high percentage of local anesthetic spread into the obturator canal, compared with the nerve stimulation-guided approach. Performing the procedure in the lithotomy position may favor cranial diffusion due to advantageous anatomical alignment. Conversely, the presence of well-developed superior fascicle of the external obturator muscle can impede drug diffusion and contribute to ONB failure. Future studies should explore approaches that directly target the external opening of the obturator canal, considering anatomical variability of the superior fascicle.

## Data Availability

The datasets used and/or analyzed during the current study are not publicly available due to ethical and privacy restrictions, but are available from the corresponding author upon reasonable request.

## Glossary

NS-INTAD: Nerve stimulation-guided interadductor approach
US-INTAD: Ultrasound-guided interadductor approach
